# Allosteric factors in the calcium/calmodulin-responsive kinase II hub domain determine selectivity of GHB ligands for CaMKIIα

**DOI:** 10.1016/j.jbc.2025.108543

**Published:** 2025-04-24

**Authors:** Stine J. Gauger, Line B. Palmelund, Yongsong Tian, Aleš Marek, Mathias R. Namini, Nane Griem-Krey, Madeline Y. Petersen, Stefanie Kickinger, Jonas S. Mortensen, Bente Frølund, Petrine Wellendorph

**Affiliations:** 1 Department of Drug Design and Pharmacology, University of Copenhagen, Copenhagen, Denmark; 2 Institute of Organic Chemistry and Biochemistry, The Czech Academy of Sciences, Prague, Czech Republic

**Keywords:** molecular pharmacology, site-directed mutagenesis, radioligand binding, homology modelling, Ca^2+^/calmodulin-dependent protein kinase II (CaMKII), pocket loop, tryptophan (Trp403)

## Abstract

The Ca^2+^/CaM-dependent protein kinase II alpha (CaMKIIα) is a highly important synaptic protein, which comprises a unique holoenzyme structure organized *via* the central hub domain. Recently, a distinct binding pocket in the CaMKIIα hub domain was identified for the endogenous neuromodulator **γ**-hydroxybutyric acid (GHB) and related synthetic analogs. Intriguingly, of the four native CaMKII isozymes, only CaMKIIα accommodates GHB ligands. Key interacting residues in CaMKIIα were revealed, but their involvement in selectivity toward the alpha variant of CaMKII has remained unresolved. Aimed at elucidating the molecular determinants for this selectivity, we here conducted binding studies to CaMKII-HEK whole-cell homogenates using two different in-house–developed GHB-related radioligands, 3-hydroxycyclopent-1-enecarboxylic acid ([^3^H]HOCPCA) and [^3^H]O-5-hydroxydiclofenac, in combination with site-directed mutagenesis. Binding to CaMKIIα with the smaller type radioligand [^3^H]HOCPCA validated key involvement of the four known residues (His395, Arg433, Arg453, and Arg469), but also revealed a role for the upper hub flexible loop containing the CaMKIIα-specific residue Trp403 (Leu in all other CaMKII isozymes) previously suggested to be involved in holoenzyme stability. Insertion of the corresponding residues (L467W/C533R) into CaMKIIβ failed to induce [^3^H]HOCPCA binding. However, with the larger type radioligand, [^3^H]O-5-hydroxydiclofenac, specific binding in CaMKII**β** (L467W/C533R) was achieved. Thus, the study confirms involvement of central binding residues and identifies the CaMKIIα flexible pocket loop as a distantly located allosteric factor in determining selectivity of GHB analogs for CaMKIIα. It sheds light on a remarkable interplay of the entire hub cavity for accommodation of ligands and corroborates GHB analogs as CaMKIIα-selective.

The Ca^2+^/CaM-dependent protein kinase II (CaMKII) is a Ser/Thr kinase involved in several Ca^2+^-signaling pathways. Four distinct, but highly related, genes (CAMK2A, CAMK2B, CAMK2D, and CAMK2G) encode the four main isozymes of CaMKII (1). CaMKIIα and CaMKIIβ are predominantly found in the brain and play an important role in synaptic plasticity underlying learning and memory (reviewed by (2, 3)). CaMKIIδ and CaMKIIγ are more ubiquitously expressed and are found in the brain, skeletal muscle, and the heart, with CaMKIIδ suggested to be involved in cardiovascular diseases (4). CaMKII has a unique holoenzyme structure composed of subunits assembled into a stacked arrangement of two donut-shaped structures (5, 6). Each subunit consists of an N-terminal catalytic kinase domain, a regulatory segment containing the binding site for Ca^2+^/CaM and central phosphorylation sites (Thr286 and Thr305/Thr306), a variable linker, and a C-terminal hub domain responsible for holoenzyme assembly (5). The hub domains of CaMKIIα and CaMKIIβ share 77% overall sequence identity (6, 7). In addition to the canonical holoenzyme assembly, a role for the hub domain in allosteric regulation of kinase activity is emerging, supported by structural work (5, 7, 8). Furthermore, the hub *via* its known fluctuations in oligomeric structure, is suggested to play a role in spreading of kinase activity (activation-triggered subunit exchange), or may occur as interholoenzyme phosphorylation without hub domain mixing (7, 9, 10, 11) potentially mechanistically involved in sustainment of memories (12, 13).

In 2021, we reported the identification of a molecularly distinct binding pocket in the hub domain of CaMKIIα, which specifically binds small molecules related to the γ-aminobutyric acid metabolite, γ-hydroxybutyric acid (GHB) (14). This work was enabled by the availability of selective and nanomolar affinity GHB analogs (15, 16, 17). GHB is endogenously present in the brain in micromolar concentrations and is used as therapeutic treatment of narcolepsy and alcohol dependence (18, 19). At therapeutic doses, GHB is known to interact with other sites in the brain, particularly. γ-aminobutyric acid subtype B receptors (18, 20). Intriguingly, GHB analogs bind exclusively to CaMKIIα, corroborated by the complete absence of binding in brain slices from *Camk2a*^*−/−*^ mice with three different ^3^H-labeled GHB analogs (radioligands), and a photoaffinity ligand, yet binding was fully preserved in *Camk2b*^*−/−*^ tissues (14). Also, radioligand binding was absent in HEK cell–expressed CaMKIIβ, γ and δ^14^. Using the high-affinity analog, 5-hydroxydiclofenac (5-HDC) (Fig. 1*A*) (17), a crystal structure of the hub domain was obtained, highlighting the key residues involved in the binding pocket. These key residues included the positively charged Arg433, Arg453, and Arg469, and His395 (14). Additionally, the crystal structure revealed a significant movement of Trp403 (Trp-flip) upon binding of 5-HDC (14). This outward placement of Trp403 has since been corroborated with other GHB ligands (21, 22). Interestingly another key tool compound, 3-hydroxycyclopent-1-enecarboxylic acid (HOCPCA) (Fig. 1*A*), does not cause a detectable Trp-flip, probably due to its small size (14). Hence, the flexible loop in the upper cavity of the identified binding pocket (the pocket loop) can present with two distinct conformations: An inward-flipped conformation with the Trp403-associated loop pointing into the hub cavity, and an outward-flipped conformation where the Trp403-associated loop is displaced outward. GHB and its analogs confer vast stabilization of the purified CaMKIIα hub domain to various extents depending on the applied analog (14, 21, 22). Yet, the functional consequence of such stabilization remains unclear, as GHB and HOCPCA were shown not to affect CaMKIIα activity (syntide-2 phosphorylation (14, 22). Intriguingly, GHB and brain-permeable analogs, such as HOCPCA, have been found to be neuroprotective in several mouse models of ischemia, pointing to a potential clinical utility in targeting the CaMKIIα hub domain (14, 23, 24).

**Figure 1:**
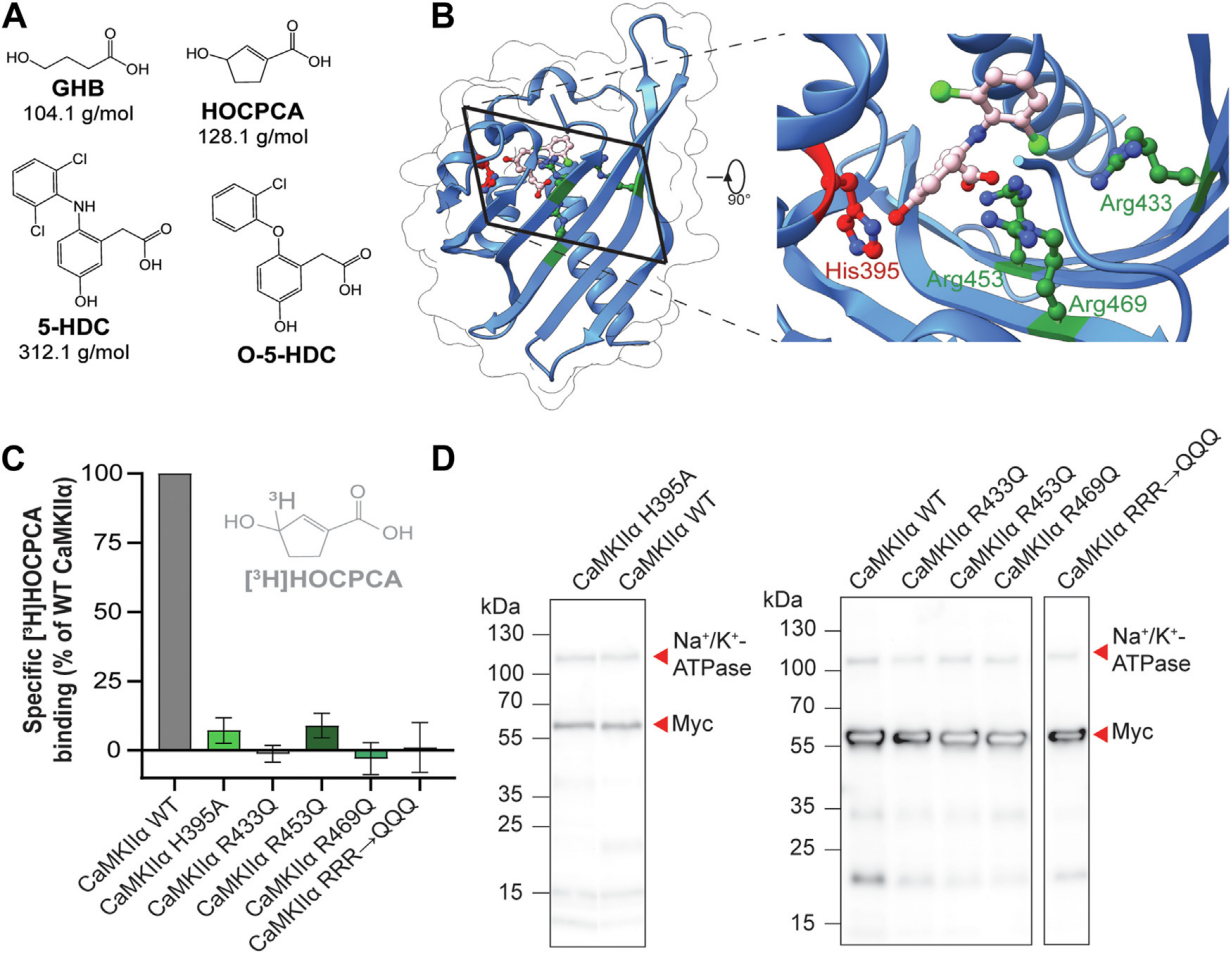
**Residues essential for [^3^H]HOCPCA binding.***A*, chemical structures of GHB ligands. *B*, crystal structure of the CaMKIIα hub domain with the four crucial residues His395 in *red*, and Arg433, Arg453, and Arg469 in *green*. Close-up view shows the position and orientation of the residues toward the inside of the binding pocket (PDB: 7REC). *C*, absence of [^3^H]HOCPCA binding to CaMKIIα binding pocket mutants; RRR→QQQ, triple mutant R433Q/R453Q/R469Q. *D*, representative western blots confirming CaMKIIα expression visualized by the C-terminal myc-tag; Na^+^/K^+^-ATPase as loading control. CaMKII, Ca^2+^/CaM-dependent protein kinase II; GHB, γ-hydroxybutyric acid; HOCPCA, 3-hydroxycyclopent-1-enecarboxylic acid.

So far, the pronounced selectivity of these small-molecule GHB ligands for CaMKIIα over CaMKIIβ, CaMKIIγ, or CaMKIIδ has remained a riddle, despite identification of interacting residues and involvement of additional factors (Trp403). Thus, this study sought to identify molecular determinants responsible for the CaMKIIα selectivity of GHB analogs by exploring specific residues both in the known binding pocket and the nearby cavity. To this end, we performed molecular modeling and site-directed mutagenesis of CaMKII isozymes. Engineered mutants were heterologously expressed in HEK293T cells and evaluated in radioligand binding assays with the standard radioligand [^3^H]HOCPCA, and a newly developed probe [^3^H]O-5-HDC based on chemical optimization of 5-HDC (17).

## Results and discussion

### Central binding residues involved in binding of GHB ligands to CaMKII**α**

The 5-HDC/CaMKIIα hub crystal structure (PDB: 7REC) revealed four key residues interacting directly with the ligand, thus constituting the core binding pocket: His395, Arg433, Arg453, and Arg469 (14) (Fig. 1*B*). These residues are oriented with the sidechains directed toward an inner cavity of the binding pocket (Fig. 1*B*), enabling electrostatic and hydrogen bond interactions with 5-HDC. Thus, to validate the importance of the identified residues, we initially evaluated [^3^H]HOCPCA binding in CaMKIIα-HEK whole-cell homogenates using site-directed mutagenesis of these four residues. Convincingly, mutation of Arg433 or Arg469 into their noncharged counterparts (R433Q and R469Q), completely abolished [^3^H]HOCPCA binding (Fig. 1*C*). For the H395A and R453Q mutants, a very minor degree of specific binding remained (7–9%). As expected, the triple mutant (RRR433/453/469QQQ) was completely binding-deficient. Western blot analysis (Fig. 1*D*) confirmed intact expression of all mutants, to an extent comparable to WT with no statistical differences detected (Fig. S1). This infers that oligomerization is likely also intact. Indeed for the W403L mutant, intact oligomerization of the hub domain has been confirmed by small-angle X-ray scattering (22). Collectively, these data validate that the four residues, originally identified in the crystal structure of 5-HDC (14), are also essential for [^3^H]HOCPCA binding, thus resembling the central binding residues for binding of GHB analogs to the CaMKIIα hub.

### Introduction of the missing arginine in CaMKII**β** and CaMKII**γ** failed to induce GHB ligand binding

**Figure 2:**
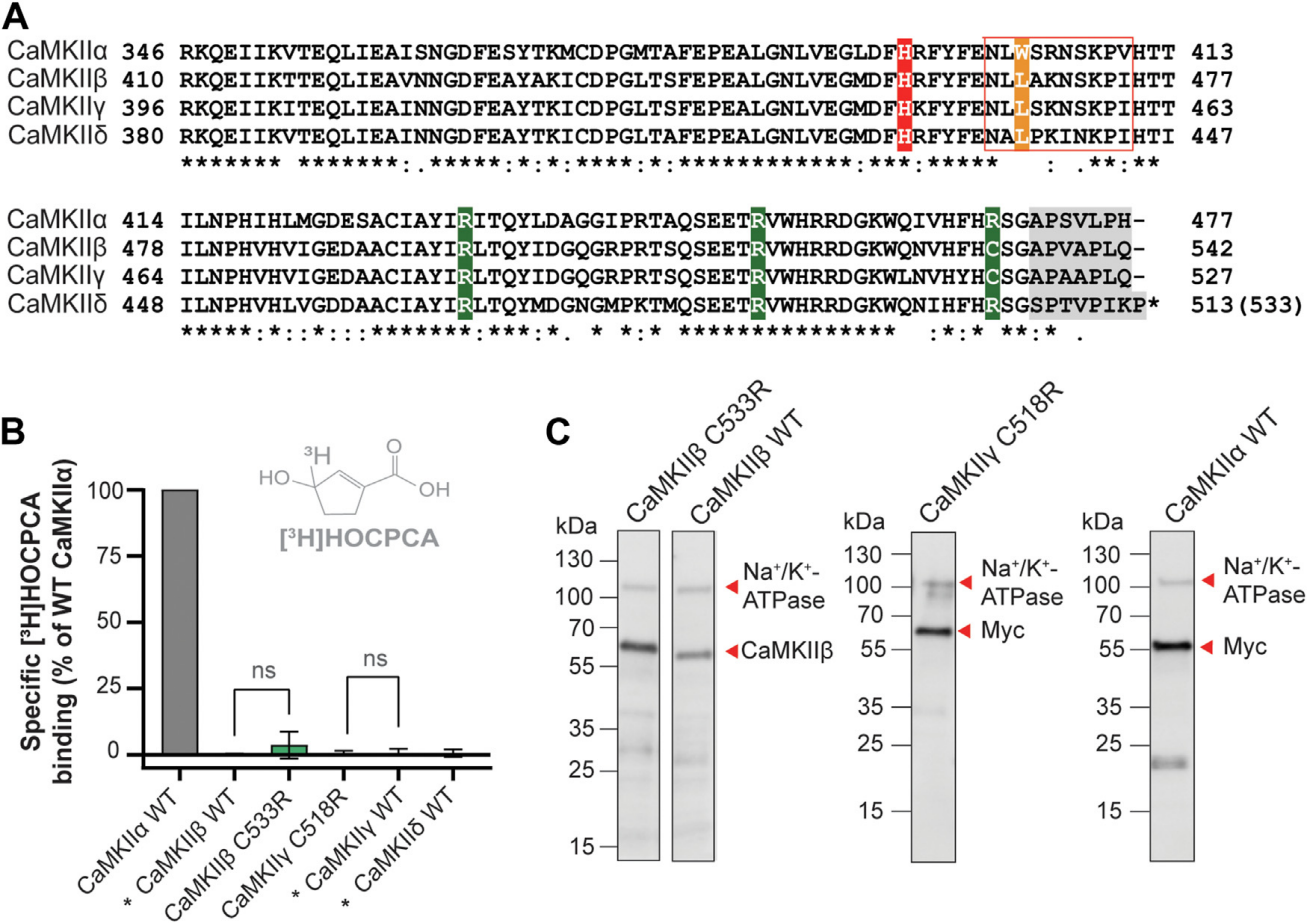
**Additional contributing factors are important for[^3^H]HOCPCA binding.***A*, sequence alignment of CaMKII rat hub domains with key molecular determinants highlighted. The *red box* signifies the pocket loop with the key residue W403 in *orange*; predicted C-tail in *gray*. *B*, specific binding of [^3^H]HOCPCA to CaMKIIβ WT, CaMKIIβ C533R (*p* = 0.2861), CaMKIIγ WT, CaMKIIγ C518R (*p* = 0.7473), and CaMKIIδ WT. ∗Data taken from (14). *C*, representative western blots confirming CaMKII expression by CaMKIIβ antibody or C-terminal myc-tag; Na^+^/K^+^-ATPase as loading control. Data were pooled from three independent experiments performed in technical triplicates and shown as mean bar graphs ± SD. Student's *t* test; significance level *p* < 0.05; ns = not significant. CaMKII, Ca^2+^/CaM-dependent protein kinase II; GHB, γ-hydroxybutyric acid; HOCPCA, 3-hydroxycyclopent-1-enecarboxylic acid.

We previously showed that [^3^H]HOCPCA and related GHB analogs display remarkable selectively for CaMKIIα (14). A simple explanation for the pronounced alpha selectivity could be the absence of one or more of the identified central binding residues. According to a multiple sequence alignment of the four CaMKII hub domains (Fig. 2*A*), among the four central binding residues (marked green and red in Fig. 2*A*), only Arg469 (CaMKIIα) differs between the CaMKII isozymes. All four residues are conserved in CaMKIIδ, whereas Arg469 is a cysteine in CaMKIIβ and CaMKIIγ (C533 and C518, respectively). Reasoning that introduction of the “missing arginine” in CaMKIIβ and CaMKIIγ might restore [^3^H]HOCPCA binding, we introduced an arginine into the corresponding position in CaMKIIβ (C533R) and CaMKIIγ (C518R) and performed radioligand binding with [^3^H]HOCPCA. As shown in Figure 2*B*, no specific binding was observed in either construct (Western blot analysis verified similar protein expression for all constructs (Fig. 2*C*)). This highlights that the mere presence of all four core binding residues is insufficient to induce GHB ligand binding and suggests additional molecular determinants to be responsible for the unique alpha selectivity.

### Importance of the CaMKII**α** pocket loop for GHB ligand binding and selectivity to CaMKII**α**

**Figure 3:**
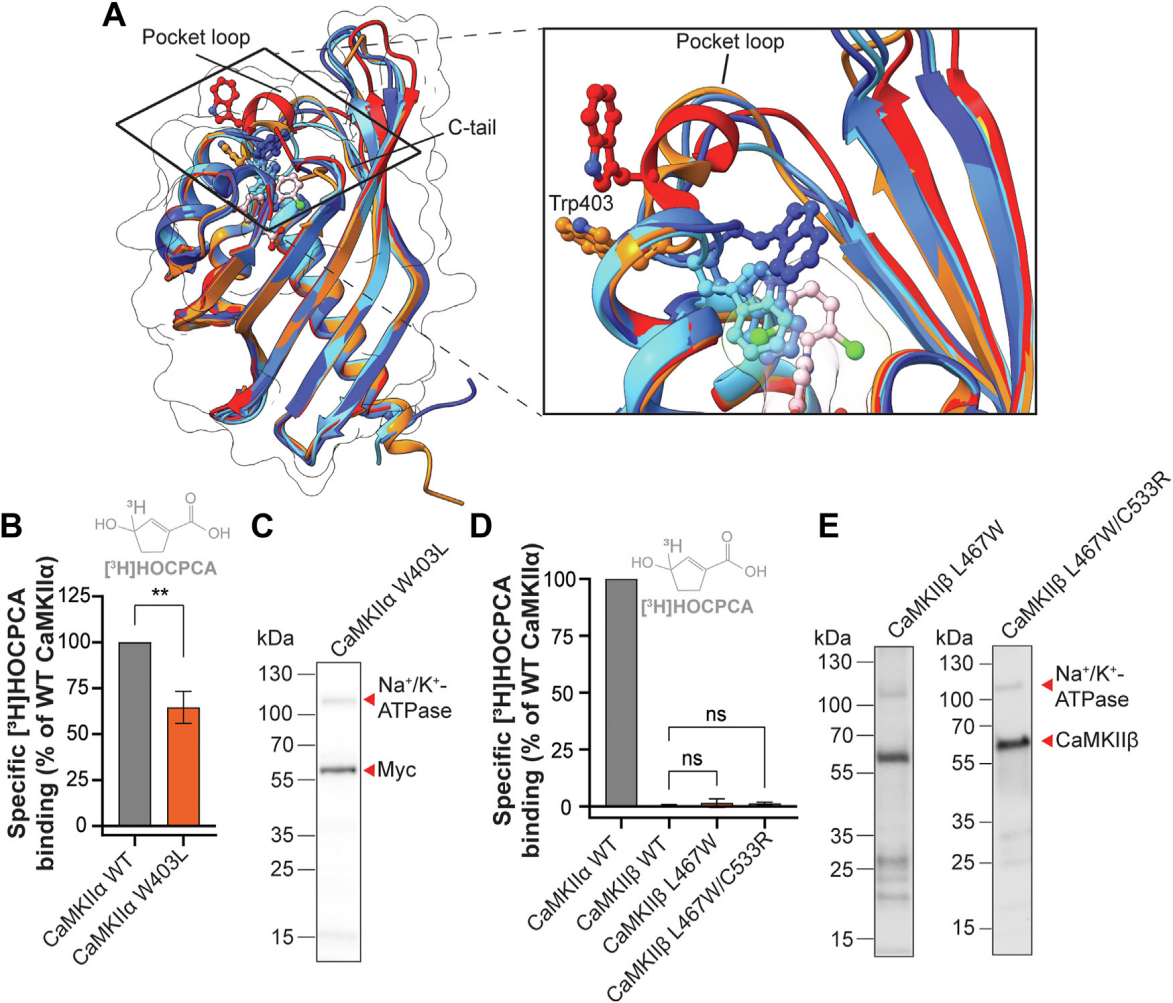
**The CaMKIIα pocket loop is important for optimal binding of [^3^H]HOCPCA.***A*, crystal structure alignment of CaMKIIα hub domains; unbound state (PDB codes: 7REC in *blue*, 3SOA in *dark blue,* and 6OF8 in *light blue*), and bound state (PDB codes: 1HKX in *orange* and 7REC in *red*), highlighting the pocket loop and the C-tail. The close-up view shows the different orientations and positions of Trp403 in the loop. *B*, specific [^3^H]HOCPCA binding to CaMKIIα W403L decreased compared to CaMKIIα WT (*p* = 0.0038). One sample *t* test significance level *p* < 0.05. *C* and *E*, Western blot validation of protein expression; Na^+^/K^+^-ATPase as loading control. *D*, no detected specific [^3^H]HOCPCA binding to CaMKIIβ WT, L490W (*p* = 0.568), and L490W/C533R (*p* = 0.6618). One-way ANOVA, *post hoc* Dunnett’s test. Significance level *p* < 0.05; ns = not significant. Data are pooled from three independent experiments performed in technical triplicates and shown as mean bar graph ± SD. CaMKII, Ca^2+^/CaM-dependent protein kinase II; GHB, γ-hydroxybutyric acid; HOCPCA, 3-hydroxycyclopent-1-enecarboxylic acid.

Next, the selectivity analysis was broadened to include additional regions and/or residues in the hub cavity that might be important for binding. For this purpose, we aligned five available crystal structures of CaMKIIα hub proteins reported to have the lowest number of unresolved residues (Table S1). The overlay revealed two highly flexible regions in the hub domain with a fairly high degree of nonconserved residues, which were located opposite to each other at the upper part of the binding cavity distant from the GHB analog binding site (Fig. 3*A*). We hypothesized that one or both of these regions might play an important role for ligand accessibility and create a favorable environment for the bound ligand. In this study, we will refer to these two regions as the pocket loop and the C-tail. In CaMKIIα, the pocket loop consists of residues between the N-terminal alpha helix and the first beta sheet (residues from Asn401 to Val410), highlighted in the sequence alignment (Fig. 2*A*).

In the structural overlay (Fig. 3*A*), large conformational flexibility was observed for Trp403 in the pocket loop, a residue unique to CaMKIIα (Fig. 2*A*). As demonstrated, this Trp403 moves substantially from the inside of the cavity to the outside upon occupancy of the pocket with, for example, 5-HDC (Fig. 3*A*) (14, 21, 22), which appears to depend on the size of the ligand and its ability to extend into the upper part of the pocket (22). Interestingly, in one reported structure (PDB 1HKX), binding of a chloride ion in the lower part of the binding cavity likewise induces a Trp-out conformation, further underlining that the flexibility of this region is influenced by ligand or ion occupancy (PDB code: 1HKX) (6). This prompted us to investigate the contribution of Trp403, first by mutating the CaMKIIα Trp into Leu (W403L), which is the residue present in the other CaMKII variants. This resulted in a slight reduction of [^3^H]HOCPCA binding in the W403L mutant (70% of WT, Fig. 3*B*) with unchanged overall protein expression (Figs. 3*C* and S1). The reduction in binding indicates that Trp403 is an additional contributing factor for binding of GHB ligands to CaMKIIα.

Despite the lack of Trp403 in CaMKIIβ, CaMKIIδ, and CaMKIIγ, crystal structure alignment revealed a similar flexible loop (Fig. S2 and Table S2). For initial studies, we explored how introduction of a Trp at the corresponding location in CaMKIIβ (L467) might affect GHB ligand binding. To this end, [^3^H]HOCPCA binding was evaluated in CaMKIIβ *via* the single mutant L467W and the double mutant L467W/C533R. Neither of these led to any noticeable radioligand binding of [^3^H]HOCPCA (Fig. 3*D*) albeit protein expression was intact (Fig. 3*E*).

### Development of [^3^H]O-5-HDC as a tool to study compound-induced CaMKII**α** conformations

**Figure 4:**
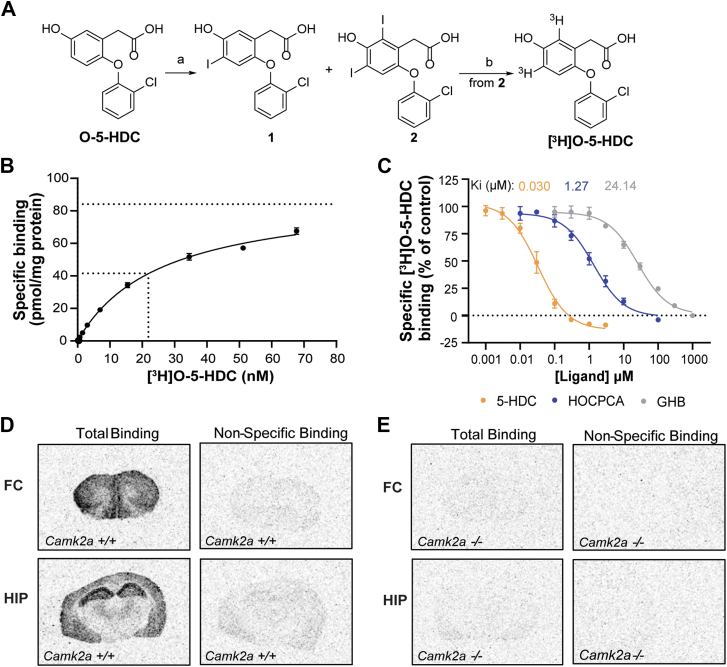
**Development and characterization of [^3^H]O-5-HDC.***A*, schematic overview of [^3^H]O-5-HDC synthesis. Reagents and conditions: a) I_2_, 30% H_2_O_2_ (w/w%) aqueous solution, H_2_O 50 °C, overnight. *B*, Pd/C, ^3^H_2_, Et_3_N, MeOH, 2 h. *B*, saturation isotherm of [^3^H]O-5-HDC; 1 mM GHB for NSB. *C*, concentration-dependent inhibition of [^3^H]O-5-HDC binding by GHB (MW = 104.1 g/mol) and analogs HOCPCA (MW = 128.1 g/mol) and 5-HDC (MW=312.1 g/mol). *B* and *C*, data are representative of at least three independent experiments performed in triplicates and shown as mean ± SD; K_i_ values are summarized in Table S4. Specific binding of [^3^H]O-5-HDC in frontal cortex (FC) and hippocampus (HIP) in brain slices from (*D*) *Camk2a* +/+ mice and (*E*) *Camk2a* −/− mice; 1 mM GHB for NSB. The autoradiograms are representative of four slices from a single experiment. 5-HDC, 5-hydroxydiclofenac; CaMKII, Ca^2+^/CaM-dependent protein kinase II; GHB, γ-hydroxybutyric acid; HOCPCA, 3-hydroxycyclopent-1-enecarboxylic acid; MW, molecular weight; NSB, nonspecific binding.

Chemically, HOCPCA (molecular weight 128.1 g/mol) and 5-HDC (molecular weight 312.1 g/mol) are representatives of two different GHB ligand classes with difference in size and binding kinetics (5-HDC has a lower off-rate than HOCPCA (14)). Moreover, the fact that they stabilize different conformations of CaMKIIα (the Trp-in and Trp-out conformations, respectively) (14), makes them interesting to compare. Thus, based on the hypothesis that additional hub dynamics involving the Trp403-containing pocket loop could impact the selectivity of GHB ligands, we aimed to develop a radioligand analog of 5-HDC that could serve as a useful tool to discern conformational effects. Since 5-HDC has been found to be chemically unstable (17) and synthesis of the dichloro analog proved troublesome, the oxygen-bridged monochloro version (O-5-HDC) was used instead (Fig. 1*A*). These compounds were found to have similar K_i_ values in CaMKIIα binding assays previously reported (K_i_ of 5-HDC = 35 nM, O-5-HDC = 27 nM) (17). As [^3^H]O-5-HDC is a novel tool, a synthesis overview is provided in Figure 4*A* and full details in the Supporting Information. Notably, two tritium isotopes are incorporated per molecule of O-5-HDC, leading to a high-specific activity of 48.2 Ci/mmol.

Before using [^3^H]O-5-HDC for addressing selectivity, assay conditions were established with native CaMKIIα in rat cortical homogenate (Fig. S3 and Tables S3 and S4). The developed assay is highly similar to the protocol for [^3^H]HOCPCA using rat cortical homogenate (25). The binding reaction followed a saturable profile (Fig. 4*B* and Table S4) and revealed a K_D_ value of 22 nM and a maximum binding capacity (B_max_) of 86.5 pmol/mg protein. This is noteworthy, as the K_D_ signifies a 10-fold improved affinity compared to [^3^H]HOCPCA (Table S4) (25, 26). Moreover, K_i_ values for GHB, HOCPCA, and 5-HDC were determined, yielding values of 24.1 μM, 1.27 μM, and 0.030 μM, respectively (Fig. 4*C* and Table S5). Interestingly, the smaller sized ligands HOCPCA and GHB showed 10-fold lower affinity in displacing [^3^H]O-5-HDC than [^3^H]HOCPCA (Table S5). Moreover, with 1 mM GHB to determine nonspecific binding (NSB), 5-HDC displayed a plateau below 0% (around −10%), suggesting a limited capacity of GHB to displace [^3^H]O-5-HDC. On the contrary, inhibition of [^3^H]HOCPCA binding by 5-HDC has previously revealed a bottom plateau around 20% (14), indicating that a proportion of bound [^3^H]HOCPCA cannot be displaced by 5-HDC. This clearly highlights small but significant probe difference in two radioligands, despite interaction with the same core residues. As such, this may reflect that different, or more, molecular interactions are at play for the slightly larger compound O-5-HDC. Alternatively, it could reflect altered levels of occupancy dependent of probe size and interaction, as 5-HDC (and presumably O-5-HDC) causes an outward-flipped movement of Trp403 which could alleviate a different hub confirmation. Indeed, [^3^H]O-5-HDC did show two times higher B_max_ than [^3^H]HOCPCA (14), which could indicate a larger proportion of binding sites or capacity for [^3^H]O-5-HDC. To confirm CaMKIIα selectivity for [^3^H]O-5-HDC as for [^3^H]HOCPCA, autoradiography on mouse brain slices using 0.09 nM [^3^H]O-5-HDC was conducted. This revealed high-density binding in frontal cortex and hippocampus (Fig. 4*D*), matching the regional binding of [^3^H]HOCPCA (27). The observed binding was displaceable by 1 mM GHB and was absent in *Camk2a*^*−/−*^ (KO) mice (Fig. 4*E*), confirming specific CaMKIIα interaction. Compared to [^3^H]HOCPCA, the use of [^3^H]O-5-HDC resulted in autoradiograms of higher quality due to the higher radioligand sensitivity.

### Successful introduction of [^3^H]O-5-HDC binding into CaMKII**β**

**Figure 5:**
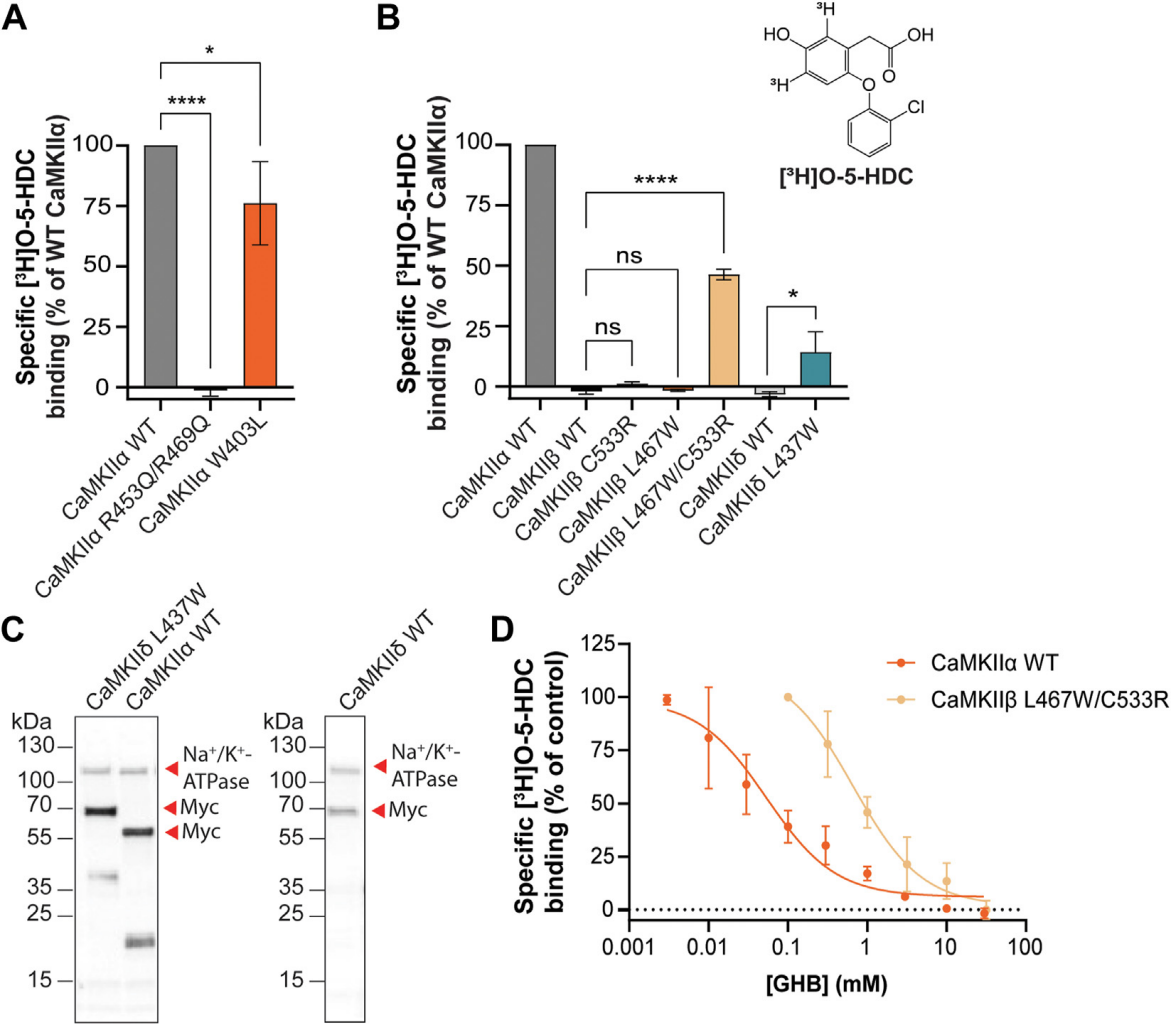
**Mutation of residue 467 (L467W) in the CaMKIIβ pocket loop promotes binding.***A*, specific binding of [^3^H]O-5-HDC to the CaMKIIα mutant R453Q/R469Q (*p* < 0.0001) and W403L (*p* = 0.0179). *B*, specific [^3^H]O-5-HDC binding to CaMKIIβ C533R (*p* = 0.2243), L467W (*p* = 0.9912), and L467W/C533R (*p* < 0.0001) and CaMKIIδ L437 W (*p* = 0.0254) mutants. *C*, Western blot validation of protein expression; Na^+^/K^+^-ATPase as loading control. *D*, concentration-dependent displacement of [^3^H]O-5-HDC binding by GHB in CaMKIIβ L467W/C533R and CaMKIIα WT. Data are pooled from three to four independent experiments performed in technical triplicates and shown as mean ± SD. One-way ANOVA, *post hoc* Dunnett’s test; significance level *p* < 0.05. 5-HDC, 5-hydroxydiclofenac; CaMKII, Ca^2+^/CaM-dependent protein kinase II; GHB, γ-hydroxybutyric acid.

With the availability of [^3^H]O-5-HDC as a conformationally selective and highly specific CaMKIIα radioligand, we revisited our mutants in the new binding assay. As expected, [^3^H]O-5-HDC binding to the mutants of the central binding residues R453Q/R469Q was still completely absent (*p* < 0.0001) (Fig. 5*A*). Furthermore, we observed a slight significant reduction in specific binding of [^3^H]O-5-HDC to W403L (75% of WT, *p* = 0.0179) (Fig. 5*A*). This was also observed for [^3^H]HOCPCA (Fig. 3*B*), confirming involvement of Trp403 as an additional factor of GHB analog binding. This coincides with slightly different K_D_ values of another GHB analog, PIPA, to purified CaMKIIα-WT *versus* CaMKIIα-W403L hub protein in surface plasmon resonance studies (1.4 μM for CaMKIIα WT and 2.8 μM for CaMKIIα-W403L) (22).

Finally, [^3^H]O-5-HDC binding was performed to the CaMKIIβ mutants, L467W, C533R, and L467W/C533R. Whereas no binding was observed for the single mutations, the double mutant L467W/C533R now presented with a significant amount of specific [^3^H]O-5-HDC binding compared to WT (*p* < 0.0001) (Fig. 5*B*), confirming that the central binding residues together with additional factors contribute to determine CaMKIIα selectivity. Further, the specific binding at CaMKIIβ L467W/C533R was displaceable by GHB in a concentration-dependent manner (IC_50_ value of 710 μM (3.17 ± 0.07) (Fig. 5*D*). However, GHB displayed a 10-fold decrease in affinity when compared to CaMKIIα WT (IC_50_ value of 61.7 μM (4.28 ± 0.20)) (Fig. 5*D*), suggesting involvement of, yet unidentified, additional residues or features. Another factor relevant for GHB analog interaction could be the size of the binding cavity. This was explored by measurement of cavity size in the 12-mer and 14-mer rat CaMKIIα and CaMKIIβ crystal structures and indeed suggest a slightly larger cavity in CaMKIIβ than CaMKIIα (Fig. S4). As such, a larger binding cavity might be less favorable for GHB analog interaction and could contribute to the observed differences in binding affinity.

Since the central binding residues are conserved in CaMKIIδ, we were intrigued to identify if introduction of the corresponding Trp in position L437 would be sufficient to induce [^3^H]O-5-HDC binding in this additional CaMKII isozyme. Curiously, introduction of the corresponding Trp mutation in CaMKIIδ (L437W) resulted in detectable [^3^H]O-5-HDC–specific binding (Fig. 5*B*) (*p* = 0.025), however to a lesser extent than CaMKIIβ L467W/C533R (14% and 46% of CaMKIIα WT-specific binding, respectively). This could be explained by the longer C-tail of CaMKIIδ, which could affect pocket accessibility dependent on the position of this flexible region in the upper part of the cavity.

Altogether, with the advent of [^3^H]O-5-HDC and carefully engineered mutations at key positions, we were able to induce GHB ligand binding into CaMKIIβ and CaMKIIδ. One reason why [^3^H]HOCPCA binding was not achieved with CaMKIIβ could reflect the chemical differences of the radioligands, that is, only O-5-HDC contains the functional groups (aromatic rings) to enable a different conformation of the pocket loop possibly as a result of Trp403 flip. As such, [^3^H]O-5-HDC might stabilize a conformation favorable for GHB analog binding and alleviate the possibility of binding to otherwise nonbinding CaMKII isozymes. Indeed, the absence of Trp403 in the CaMKIIα pocket decreases protein thermal stability (22) and the pocket loop was previously suggested to mediate an interaction between the kinase domain and the hub domain in a docked state, which highlights a potential, yet unclear, contribution to physiological CaMKIIα function (7). Additionally, with the decreased affinity observed in the CaMKIIβ mutant compared to CaMKIIα, [^3^H]HOCPCA binding may simply not be strong enough to be detected due to its lower affinity and faster off-rate. Thus, the molecular determinants for GHB ligand selectivity are not only driven by the four central binding residues in the binding pocket but also by overall hub dynamics, signified by interactions with flexible regions located in the upper part of the hub cavity. Finally, additional factors such as other parts of the flexible region or the C-tail may impact selectivity.

## Conclusion

In summary, site-directed mutagenesis studies confirmed the importance of previously identified interacting residues for two different GHB radioligands, as well as additional probe-dependent molecular determinants in the flexible upper hub cavity. As such, this study highlights the interplay of crucial interacting residues and additional factors to determine CaMKIIα selectivity. The novel radioligand, [^3^H]O-5-HDC permitted induction of binding in the otherwise nonbinding isozyme, CaMKIIβ, and to a smaller extent, CaMKIIδ. We conclude that larger type GHB analogs such as O-5-HDC are more likely to form stabilizing interactions than smaller type ligands such as GHB and HOCPCA by compensating for the Trp403 movement, which alleviated the possibility of binding in CaMKIIβ and CaMKIIδ mutant. These findings corroborate the CaMKII alpha selectivity of chemically diverse GHB analogs. In contrast to standard nonselective CaMKII ligands (*e.g.*, CN21, AS100105 or KN93) (28), it highlights the potential of GHB analogs for targeted CaMKIIα drug discovery.

## Experimental procedures

### Compounds and radioligands

GHB sodium salt was purchased from Sigma-Aldrich. HOCPCA sodium salt and 5-HDC were prepared as reported (14, 17) (Fig. 1*A*). [^3^H]HOCPCA (spec. activity 28.6 Ci/mmol) was produced in-house as described (25). [^3^H]O-5-HDC (spec. activity 48.2 Ci/mmol) was produced as described in Supporting Information.

### Plasmids and mutants

For transfections we employed the following plasmids (all from Origene): pCMV6-CaMKIIa-Myc-DDK, (#RR201121), pCMV6-CaMKIIg-Myc-DDK (#RR207416), and pCMV6-CaMKIId-Myc-DDK. The pCAGG-CaMKIIb-pPGK-tdTOMATO plasmid for CaMKIIβ was a kind gift from Dr G. van Woerden, and has been described previously (29). The c-myc epitope tag is localized at the C terminus of CaMKII constructs. Site-directed mutagenesis and verification of the sequences (rat) was performed by GenScript. Intact expression of mutants was validated by Western blot analysis as detailed in Supporting Information.

### Radioligand binding assays

#### Recombinant CaMKII expression

Preparation of whole-cell homogenates including cell-culturing details and preparation of cortical membrane homogenates are detailed in the Supporting Information.

Recombinant HEK293T whole-cell homogenate equilibrium binding experiments were performed similar to that described previously (14), using a 48-well assay format. Briefly, whole-cell homogenate (100–150 μg protein per well) and either 40 nM [^3^H]HOCPCA or 10 nM [^3^H]O-5-HDC were incubated in a total volume of 400 μl binding buffer (50 mM KH_2_PO_4_, pH 6.0) for 1 h on ice. NSB was determined using 16 to 30 mM GHB. The binding reaction was terminated by precipitation with ice-cold acetone (1 h at −20 °C), and radioligand-bound protein was separated from free radioligand by vacuum-filtration through glass fibre (GF/C) filters. Counts (disintegrations per minute [DPM]) were detected by liquid scintillation counting (14). Counts for CaMKIIα WT typically ranged from 2000 to 6000 DPM with [^3^H]HOCPCA and 1800 to 5000 DPM with [^3^H]O-5-HDC, slightly lower for the CaMKIIβ C657R/L790W mutant. All experiments were performed in technical triplicates using at least three different batches of HEK293T cell homogenates.

#### Native CaMKIIα binding

Characterization of [^3^H]O-5-HDC binding to native CaMKIIα (rat cortical homogenate) was carried out using a 96-well format assay based on a protocol very similar to [^3^H]HOCPCA (25).For equilibrium binding, incubation of 10 to 15 μg protein on ice for 60 min with 5 nM [^3^H]O-5-HDC in a 50 mM KH_2_PO_4_, pH 6.0 buffer (total volume of 200 μl) was used. Binding was found to be linear with 0 to 25 ug total protein/well. Kinetics was assessed with association and dissociation curves using time points 1 to 120 min, and [^3^H]O-5-HDC saturation using concentrations of 0.5 to 100 nM. In all experiments, NSB was determined with 1 mM GHB. Protein-ligand bound complexes were collected by rapid filtration through GF/C filter plates and counts per minute measured in a TopCount NXT Microplate Scintillation counter (PerkinElmer) with 3 min counting per well. For saturation experiments, counts per minute were converted to DPM *via* a standard quench curve. Check of exact radioligand concentrations were determined with the TriCarb and no ligand depletion was observed.

*In vitro* autoradiography was performed on 12-μm thick coronal mouse brain sections from adult male *Camk2a* (*Camk2*^*atm3Sva*^, MGI: 2389262) (−/−) or corresponding litter mates (+/+) backcrossed in the C57BL/6j background. Experiments were performed as previously described (14) using 0.09 nM [^3^H]O-5-HDC.

### Data analysis and statistics

Data analysis was performed on pooled, normalized data using GraphPad Prism 8 (GraphPad Software Inc; https://www.graphpad.com/) as further detailed in Supporting Information. For statistical analysis, a Student's *t* test or one-way ANOVA followed by Dunnett’ multiple comparison test for comparison of mean between groups was used, as specified in the figure legends. In a few instances, a one sample *t* test with a hypothetical mean value of 100 representing normalized specific binding of WT CaMKIIα was used.

### Computational modeling

Protein visualization and alignment was performed in ChimeraX 1.7.1. The protein structures were loaded and single subunits from each PDB file were sequentially aligned using the MatchMaker extension of Chimera (30) X. The pairwise sequence alignment, constructed by MatchMaker to guide the protein superposition utilizes the Needleman–Wunsch algorithm for global and local alignment. The superposition of the listed CaMKIIα hub domains was aligned with RMSD scores ranging from 0.969 Å to 1.461 Å. Estimation of CaMKII cavity size was performed using the KVFinder (31) plugin in ChimeraX, with default setting except for “outer probe radius” which was set at 5 Å. All protein illustrations were prepared in Chimera X (32). Multiple sequence alignment was performed with Clustal Omega (EMBL-EBI).

## Data availability

This article contains no generated datasets.

## Supporting information

This article contains supporting information (14, 16, 17, 33, 34, 35, 36).

## Conflict of interest

The authors declare that they have no conflicts of interest with the contents of this article.
